# Supplementary material for: Resolving the full spectrum of human genome variation using Linked-Reads
Source: Genome Res. 2019 Apr;29(4):635–45. doi: 10.1101/gr.234443.118 (PMC6442396; doi:10.1101/gr.234443.118)
Supplement: Supplemental Material [file supp_gr.234443.118_Supplemental_longranger-2.2.zip › longranger-2.2.tar.bz2!/lr2.2-public/lib/stan/cmdstan/stan/doc/api/html/classstan_1_1_vector_view_mvt.html]

Stan: stan::VectorViewMvt< T, is\_array, throw\_if\_accessed > Class Template Reference


|  |  |
| --- | --- |
|  | Stan  2.5.0  probability, sampling & optimization |


- Main Page
- Related Pages
- Namespaces
- Classes
- Files

- Class List
- Class Index
- Class Hierarchy
- Class Members

All Classes Namespaces Files Functions Variables Typedefs Enumerations Enumerator Friends Macros Pages

- stan
- VectorViewMvt

Public Types |
Public Member Functions |
List of all members

stan::VectorViewMvt< T, is\_array, throw\_if\_accessed > Class Template Reference

`#include <traits.hpp>`

|  |  |
| --- | --- |
| Public Types | |
| typedef scalar\_type\_pre< T >::type | matrix\_t |
|  | |

|  |  |
| --- | --- |
| Public Member Functions | |
|  | VectorViewMvt (matrix\_t &m) |
|  | |
|  | VectorViewMvt (std::vector< matrix\_t > &vm) |
|  | |
| matrix\_t & | operator[] (int i) |
|  | |

## Detailed Description

### template<typename T, bool is\_array = stan::is\_vector\_like<typename stan::math::value\_type<T>::type>::value, bool throw\_if\_accessed = false> class stan::VectorViewMvt< T, is\_array, throw\_if\_accessed >

Definition at line 514 of file traits.hpp.

## Member Typedef Documentation

template<typename T, bool is\_array = stan::is\_vector\_like<typename stan::math::value\_type<T>::type>::value, bool throw\_if\_accessed = false>

typedef scalar\_type\_pre<T>::type stan::VectorViewMvt< T, is\_array, throw\_if\_accessed >::matrix\_t

Definition at line 516 of file traits.hpp.

## Constructor & Destructor Documentation

template<typename T, bool is\_array = stan::is\_vector\_like<typename stan::math::value\_type<T>::type>::value, bool throw\_if\_accessed = false>

|  |  |  |  |  |  |
| --- | --- | --- | --- | --- | --- |
| stan::VectorViewMvt< T, is\_array, throw\_if\_accessed >::VectorViewMvt | ( | matrix\_t & | *m* | ) |  |

inline

Definition at line 518 of file traits.hpp.

template<typename T, bool is\_array = stan::is\_vector\_like<typename stan::math::value\_type<T>::type>::value, bool throw\_if\_accessed = false>

|  |  |  |  |  |  |
| --- | --- | --- | --- | --- | --- |
| stan::VectorViewMvt< T, is\_array, throw\_if\_accessed >::VectorViewMvt | ( | std::vector< matrix\_t > & | *vm* | ) |  |

inline

Definition at line 520 of file traits.hpp.

## Member Function Documentation

template<typename T, bool is\_array = stan::is\_vector\_like<typename stan::math::value\_type<T>::type>::value, bool throw\_if\_accessed = false>

|  |  |  |  |  |  |
| --- | --- | --- | --- | --- | --- |
| matrix\_t& stan::VectorViewMvt< T, is\_array, throw\_if\_accessed >::operator[] | ( | int | *i* | ) |  |

inline

Definition at line 522 of file traits.hpp.

---

The documentation for this class was generated from the following file:

- src/stan/meta/traits.hpp


---

[ Stan Home Page ]

*© 2011–2014,
Stan Development Team.*
